# Domain-autonomous MKLP1 modules divergently regulate mitotic fidelity and polyploidy in a holocentric system

**DOI:** 10.1016/j.isci.2026.117149

**Published:** 2026-10-01

**Authors:** Bing Zhang, Xiao-Ning Liu, Mei-Ting Song, Meng-Fan Qin, Shu-Shu Tian, Yu Lin, Dan-Dan Li, Lun-Guang Yao, Camilo Ayra-Pardo, Yun-Chao Kan

**Affiliations:** 1 Henan Key Laboratory of Insect Biology, Henan International Joint Laboratory of Insect Biology, College of Life Science, Nanyang Normal University, 1638 Wolong Road, Nanyang, Henan 473061, China; 2 CIIMAR/CIMAR LA, Interdisciplinary Centre of Marine and Environmental Research, University of Porto, Terminal de Cruzeiros Do Porto de Leixões, Avda. General Norton de Matos S/n, 4450-208 Matosinhos, Portugal; 3 School of Life Science and Technology, Henan Institute of Science and Technology, 90 East of Hualan Avenue, Xinxiang, Henan 453003, China

**Keywords:** *Bombyx mori*, cytokinesis, MKLP1, spindle assembly, mitosis

## Abstract

While the kinesin MKLP1 is a well-established regulator of cytokinesis, whether its individual domains possess autonomous functions has remained unexplored. Here, using the holocentric *Bombyx mori* BmN4 system, we show that isolated head, stalk, and tail domains of MKLP1 exhibit distinct subcellular localization patterns and exert divergent control over mitosis. These domains differentially regulate cell-cycle progression, with the head and stalk inducing G2/M arrest, whereas the tail, and to a lesser extent the head, promotes polyploidization. Despite these divergent outcomes, all three domains converge on a shared pathological activity, aberrantly recruiting γ-tubulin, disrupting spindle organization, and compromising chromosome segregation. The domains also exert opposing effects on EdU incorporation, with the head suppressing EdU labeling and the stalk and tail enhancing it. Together, our findings redefine MKLP1 as a composite platform of regulatory modules, whose uncoupling reveals a principle of kinesin organization with broad implications for mitotic robustness and genomic stability.

## Highlights

• MKLP1 regulate mitotic fidelity and ploidy via head, stalk, and tail effects
• All MKLP1 domains aberrantly recruit γ-tubulin and disrupt spindle organization
• Domain-level perturbation uncouples cell-cycle arrest from DNA synthesis
• A holocentric system exposes latent regulatory activities of mitotic kinesins

## Graphical abstract

**Figure:**
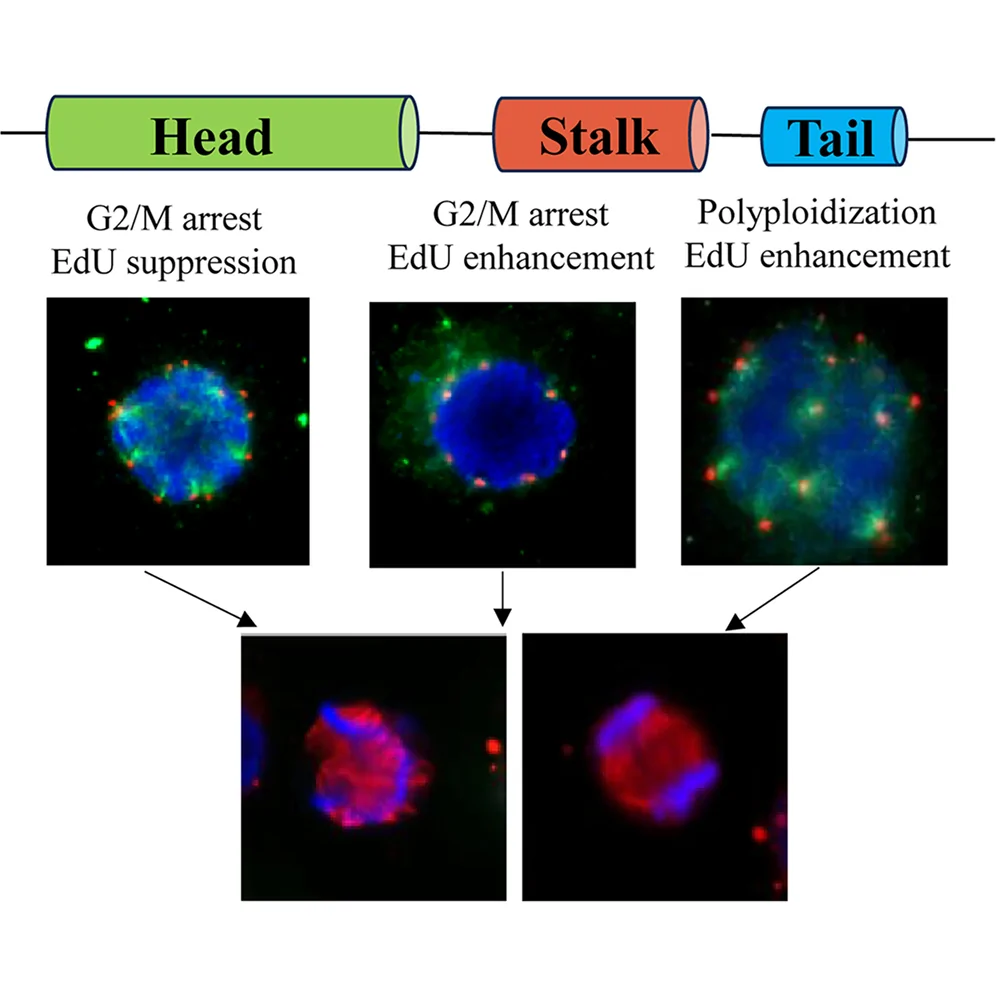

## Teaser

Molecular biology; Evolutionary biology; Cell biology; Structural biology

## Introduction

Mitosis is a fundamental and tightly regulated process crucial for cellular propagation, development, and tissue homeostasis in eukaryotes.^1^^,^^2^ Accurate chromosome segregation during cell division is critical for maintaining genomic stability, and failures in this process are hallmarks of diseases such as cancer.^3^^,^^4^ This fidelity is governed by the mitotic spindle, a dynamic microtubule-based structure whose assembly and function depend on motor proteins of the kinesin superfamily.^5^^,^^6^^,^^7^ Kinesins are modular ATP-driven motors composed of distinct structural domains, yet a central unresolved question is whether these domains merely act as inseparable components of an integrated machine or possess autonomous regulatory capacities that can influence mitotic outcomes.

MKLP1 (mtotic kinesin-like protein 1) exemplifies this modular architecture. It is a conserved kinesin-6 motor best known for its essential role in cytokinesis, where it localizes to the central spindle and mid-body to promote furrow ingression and abscission.^7^^,^^8^ However, accumulating evidence indicates that MKLP1 functions extend beyond cytokinesis.^9^ The protein comprises an N-terminal motor (head) domain, a central stalk region, and a C-terminal tail domain that mediates protein interactions, yet our understanding of how these domains contribute autonomously to mitotic regulation remains limited. Current models largely derive from studies of the intact holocomplex, leaving unresolved whether individual domains encode latent regulatory activities that are normally constrained within the full-length motor.

Importantly, for MKLP1, domain-specific functions have largely been inferred from studies of the intact centralspindlin complex. The motor (head) domain drives microtubule plus-end motility and contributes to mid-body organization,^10^^,^^11^ the stalk mediates binding to the CYK4 subunit to confer structural flexibility,^9^ and the tail recruits regulatory factors such as Plk1 to coordinate abscission.^12^ Critically, however, these roles reflect the domains’ integrated activities within the native holocomplex rather than their autonomous regulatory potential. As a result, how individual MKLP1 domains independently influence cell-cycle checkpoints, spindle assembly, and ploidy, and how their dysregulation contributes to mitotic failure, remains unresolved. This gap is underscored by observations that domain-specific perturbations of MKLP1 in mammalian cells lead to severe phenotypes such as multinucleation^10^ and chromosomal instability,^3^^,^^13^ highlighting the broader relevance of dissecting domain-level contributions to mitotic fidelity.

Studies in other kinesin families have begun to reveal that individual domains can exert specialized control over spindle dynamics and checkpoint regulation,^14^^,^^15^^,^^16^^,^^17^^,^^18^ and MKLP1 itself is subject to complex intramolecular and partner-mediated regulation. For example, binding of the CYK4 subunit reconfigures MKLP1 to optimize antiparallel microtubule bundling,^9^^,^^11^^,^^15^ while autoinhibitory interactions involving kinesin tail domains are well-established in other kinesin families.^19^ Together, these findings raise the fundamental, unresolved question: do the structural domains of MKLP1 merely function as inseparable components of a unified motor, or do they encode inherent, autonomous regulatory capacities that are normally constrained within the intact, full-length protein? Testing this “domain autonomy” hypothesis is crucial for understanding the modular evolution and functional regulation of kinesins. Furthermore, the complex buffering mechanisms present in canonical monocentric cell systems may mask these latent activities.

To directly address this question, we employed the silkworm *Bombyx mori* BmN4 cell line, which possesses holocentric chromosomes and assembles its mitotic spindle through a distinctive balance of chromatin-driven and centrosome-associated microtubule nucleation pathways.^20^^,^^21^^,^^22^ This architectural context renders the system exquisitely sensitive to perturbations in the spatial control of microtubule nucleation, thereby amplifying phenotypes and enabling the detection of domain-specific disruptive activities that might otherwise remain cryptic in more buffered, monocentric systems. We therefore systematically dissected the head, stalk, and tail domains of *B. mori* MKLP1 (BmMKLP1), examining their localization, effects on cell-cycle progression and ploidy, impact on spindle organization, and consequences for cellular proliferation. Our findings reveal that MKLP1 domains function as autonomous regulatory modules whose uncoupling profoundly alters mitotic organization and outcomes, thereby redefining BmMKLP1 from a unitary cytokinetic motor into a modular platform of regulated, and often latent, functional activities.

## Results

### Predictive structural and evolutionary analysis defines three biophysically distinct modules in BmMKLP1

To elucidate the modular organization of BmMKLP1, we integrated conserved domain annotations with predictive structural analyses. The 868 amino acid (aa) protein comprises three discrete modules: an N-terminal kinesin motor domain (aa 28–429), a central Smc-like coiled-coil region (aa 502–662), and a C-terminal tail (aa 663–868) containing a predicted Arf6-binding motif (aa 722–829) (Figure 1A). These boundaries define the three operational constructs (head, stalk, tail), which were cloned as GFP-fusion proteins under the OplE2 promoter in a pIZ/V5-His derivative for functional dissection. Coiled-coil prediction revealed a high-probability segment (aa ∼500–700) that encompasses and extends beyond the annotated stalk, supporting a dominant helical scaffold linking the motor and tail (Figure 1B). Intrinsic disorder and binding-propensity profiling further resolved the protein into biophysically distinct regions: the motor domain displayed low disorder, the coiled-coil stalk exhibited intermediate disorder, and the C-terminal tail was predominantly disordered and enriched in predicted disordered binding regions, with a localized reduction in disorder precisely coinciding with the Arf6-binding motif (Figure 1C). Consistent with these biophysical distinctions, evolutionary analysis of MKLP1 orthologs revealed strong sequence conservation of the motor domain across insects, arthropods, and vertebrates (Figure 1D). The central coiled-coil region displayed moderate conservation, characterized by preserved length and positional constraints rather than strict sequence identity. In contrast, the C-terminal tail showed substantial divergence. Together, these analyses underscore a conserved core motor-scaffold architecture and highlight the tail as a potential locus for lineage-specific regulatory adaptation, providing a structural rationale for testing the functional autonomy of individual BmMKLP1 domains.

**Figure 1:**
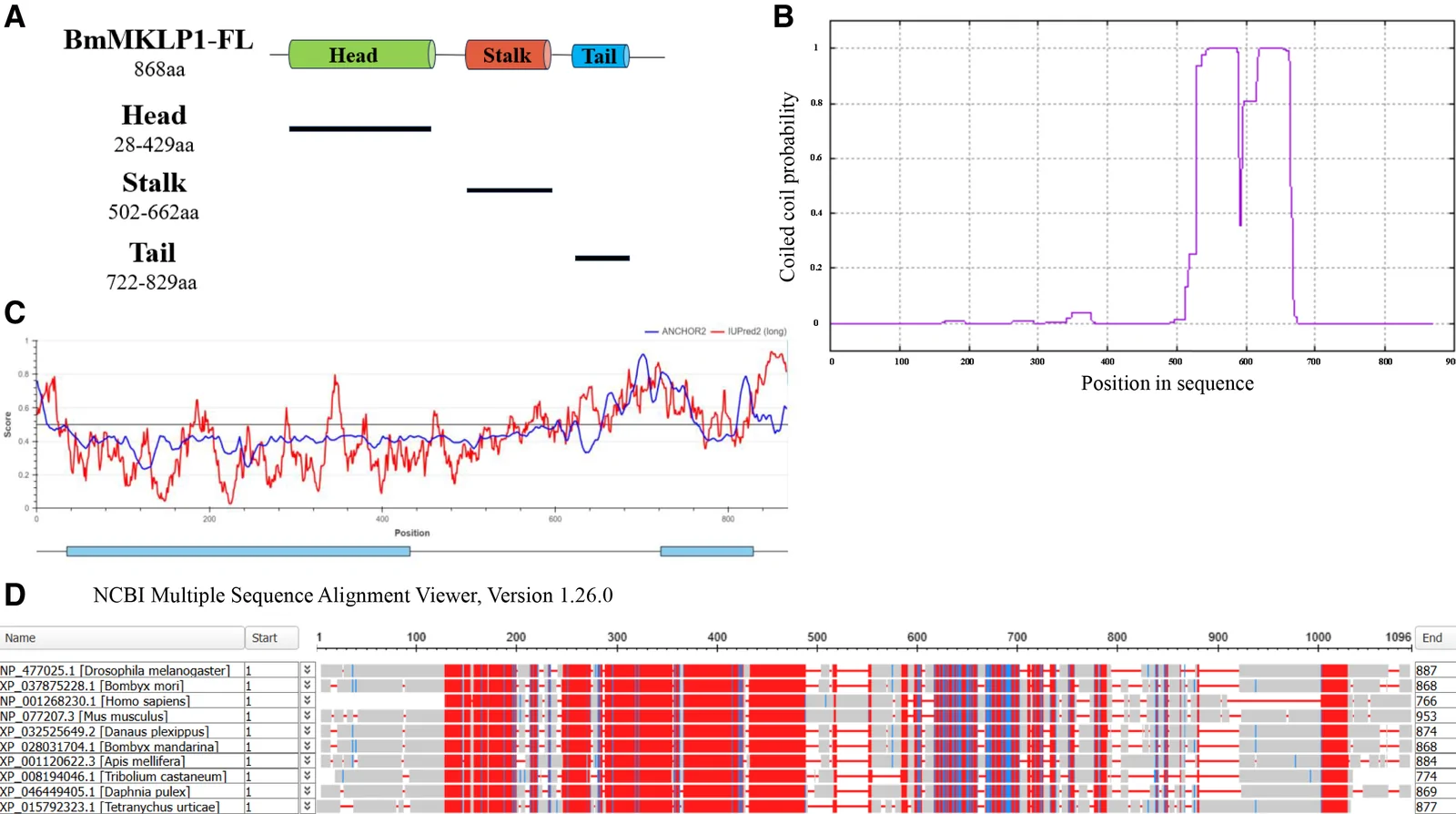
Predictive structural and evolutionary analysis defines three biophysically distinct modules in BmMKLP1 (A) Schematic of full-length BmMKLP1 (868 aa) based on the reference sequence (GenBank: XP_037875228.1), showing the KIF23-like motor (head, aa 28–429), Smc-like coiled-coil (stalk, aa 502–662), and C-terminal tail containing a predicted Arf6-binding motif (aa 722–829). (B) Coiled-coil probability profile generated by MARCOIL^23^(Gabler et al., 2020; https://toolkit.tuebingen.mpg.de/tools/marcoil). A high-probability region (probability ∼1) spanning approximately aa 500–700 supports the Stalk as a dominant coiled-coil scaffold. (C) Intrinsic disorder and disordered binding region predictions generated using IUPred2 (red) and ANCHOR2 (blue) with IUPred2A^24^(Erdős & Dosztányi, 2020; https://iupred2a.elte.hu/). The head domain exhibits low disorder (score <0.2), the stalk shows intermediate disorder, and the tail is highly disordered, with a pronounced disordered binding region corresponding to the predicted Arf6-binding motif. (D) Multiple sequence alignment of BmMKLP1 with orthologs from representative metazoan species, visualized using the NCBI Multiple Sequence Alignment Viewer (v.1.26). Strong sequence conservation across the motor domain contrasts with positional conservation in the coiled-coil region and pronounced divergence in the tail.

### The BmMKLP1 domains exhibit divergent nuclear and cytoplasmic localization

To determine whether the biophysically distinct BmMKLP1 modules encode intrinsic targeting information, we expressed GFP-tagged versions of the full-length, head, stalk, and tail domains in BmN4 cells. Fluorescence microscopy revealed clearly distinct subcellular localization patterns among the domains (Figure 2A), in stark contrast to the diffuse cytoplasmic distribution of BmMKLP1-full length. The head domain was diffusely distributed throughout both the cytoplasm and nucleus, whereas the stalk and tail domains accumulated predominantly in the nucleus. Within the nucleus, these domains exhibited divergent behaviors: the stalk domain formed discrete nuclear puncta, whereas the tail displayed a more homogeneous nucleoplasmic distribution. The formation of these stark-specific nuclear puncta, which correlates with subsequent functional defects, suggests they represent abnormal oligomeric assemblies rather than inert aggregates, a possibility we further address in the discussion. Expression of each construct was confirmed by western blot analysis, verifying the production of GFP-tagged head, stalk, and tail domains at their expected molecular weights of 73.4, 44.9, and 37.9 kDa, respectively (Figure 2B). Together, these observations demonstrate that uncoupling BmMKLP1 into its constituent domains reveals autonomous and distinct subcellular targeting behaviors.

**Figure 2:**
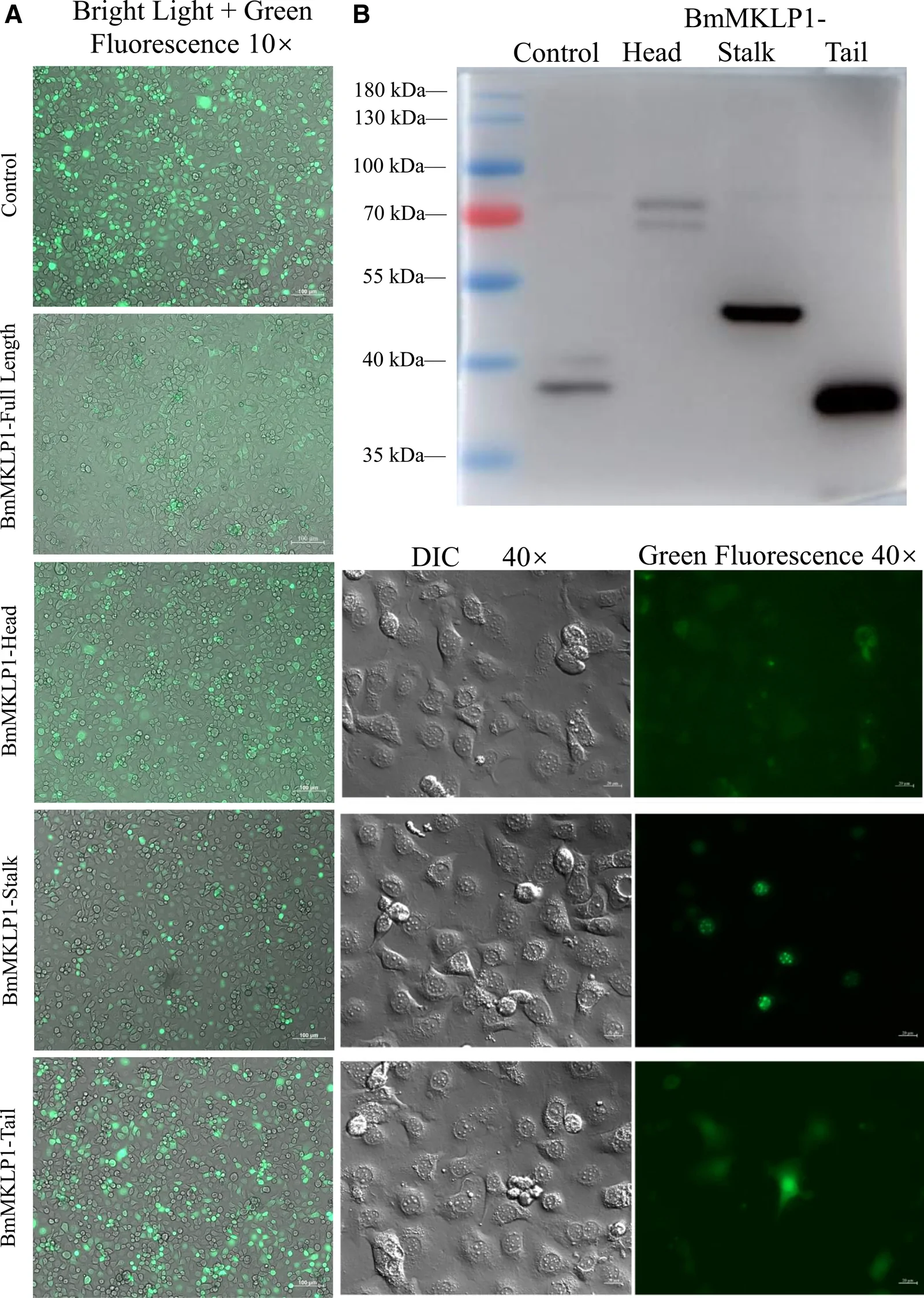
The BmMKLP1 domains exhibit divergent nuclear and cytoplasmic localization (A) Subcellular localization of transiently expressed GFP-tagged domains (green) in BmN4 cells. Representative merged bright-field and GFP fluorescence images acquired at 10×, scale bars, 100 μm, BmN4 cells expressing the indicated constructs were imaged by differential interference contrast (DIC) microscopy (left) to show cell morphology and by fluorescence microscopy (right) to detect GFP-tagged proteins. All images were acquired with a 40× objective lens. Scale bars, 20 μm. (B) Western blot analysis of protein extracts from transfected BmN4 cells probed with an anti-GFP antibody (1:1,000, Proteintech, Wuhan, China), confirming the production of each GFP-tagged domain at its expected molecular weight. The “control” lane corresponds to the cycle 3 GFP-bleo fusion protein (∼39–40 KDa) expressed from the pIZT/V5-His vector. A representative blot from three independent experiments is shown. The PageRuler Prestained Protein Ladder (10–180 kDa; Thermo Fisher Scientific, Waltham, MA, USA) was used as a molecular weight marker.

### The head and stalk domains induce G2/M arrest while the tail promotes polyploidization

To define the autonomous contributions of individual BmMKLP1 domains to cell cycle progression and genome stability, we conducted flow cytometric analysis. This revealed a functional split among domains: expression of the head or stalk domain significantly induced the G2/M phase population and reduced the S phase fraction, whereas the tail domain did not alter cell cycle phase distribution but specifically and significantly increased polyploidization (Figures 3A and 3B). Notably, the head domain also promoted a significant, though less pronounced, increase in polyploidy (Figures 3A and 3C), while the stalk domain had no effect on polyploidization. As a critical control, overexpression of BmMKLP1-full-length also induced G2/M arrest and increased polyploidy, closely resembling the effects of the isolated head domain. Together, these results indicate that the head and stalk domains primarily induce G2/M arrest, whereas the tail domain promotes polyploidization, with a secondary contribution from the head domain. Importantly, the similarity between the full-length protein and the head domain suggests that the motor activity is the primary driver of the mitotic disruption observed upon BmMKLP1 overexpression, while the distinct phenotype of the tail domain reveals a latent, specialized function that is normally constrained in the intact protein.

**Figure 3:**
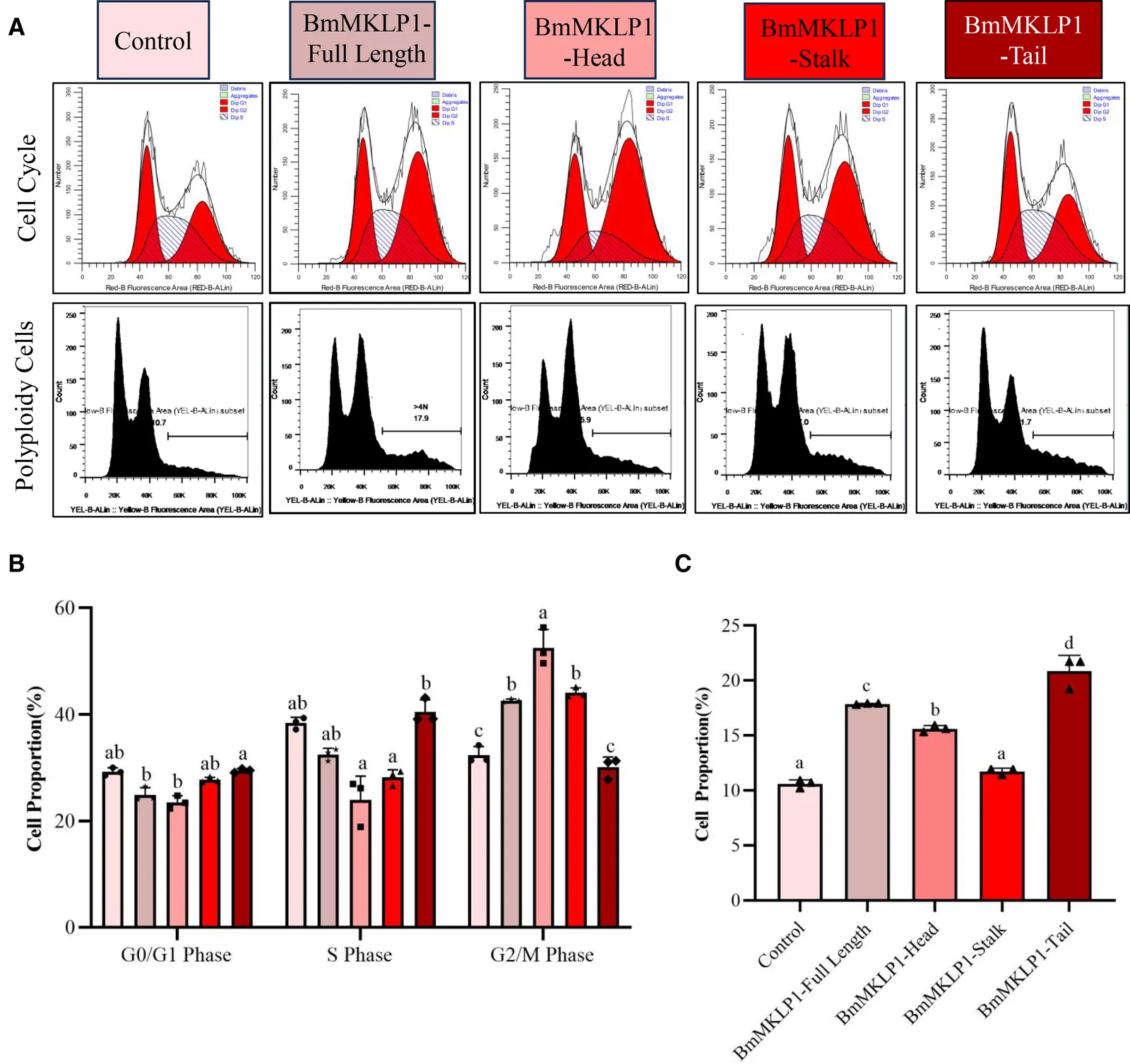
The head and stalk domains induce G2/M arrest, whereas the tail promotes polyploidization (A) Representative flow cytometry histograms showing DNA content profiles (top) and polyploidy distributions (bottom) for control cells and cells expressing GFP-tagged BmMKLP1—full-length, head, -stalk, or -tail. Cell cycle phases (G0/G1, S, and G2/M) were determined by model-based fitting of propidium iodide (PI) fluorescence intensity histograms. Polyploidy histograms depict cells with DNA content exceeding 4 N. (B) Quantification of cell cycle distribution under each condition. Bars represent the mean ± SEM percentage of cells in G0/G1, S, and G2/M phases from at least three independent experiments. Different lowercase letters indicate significant differences (one-way ANOVA followed by Tukey’s HSD, *p* < 0.05). (C) Percentage of polyploid cells under each condition. Bars represent mean ± SEM, with statistical significance assessed as in (B).

### Distinct BmMKLP1 domains promote ectopic γ-tubulin recruitment and aberrant spindle organization

To assess the capacity of individual BmMKLP1 domains to perturb spindle assembly, we examined γ-tubulin and α-tubulin organization in transfected BmN4 cells. Immunofluorescence analysis showed that control cells displayed a defined spindle architecture with no more than four γ-tubulin foci, whereas overexpression of BmMKLP1-full-length moderately increased γ-tubulin foci. Strikingly, expression of any isolated BmMKLP1 domain markedly amplified this effect, resulting in more than eight ectopic γ-tubulin foci per cell (Figure 4). This shared phenotype indicates that each domain retains an intrinsic ability to disrupt the spatial control of microtubule nucleation when expressed independently. In the holocentric BmN4 system, where centrosome-mediated microtubule organization is atypical and chromatin-associated nucleation plays a prominent role, this disruption manifested as widespread ectopic microtubule nucleation. Consistently, α-tubulin staining revealed that these ectopic γ-tubulin foci supported the formation of multi-polar microtubule arrays organized around scattered chromatin masses. Together, these results demonstrate that all three BmMKLP1 domains can override normal spatial constraints of spindle assembly by promoting γ-tubulin recruitment to ectopic sites.

**Figure 4:**
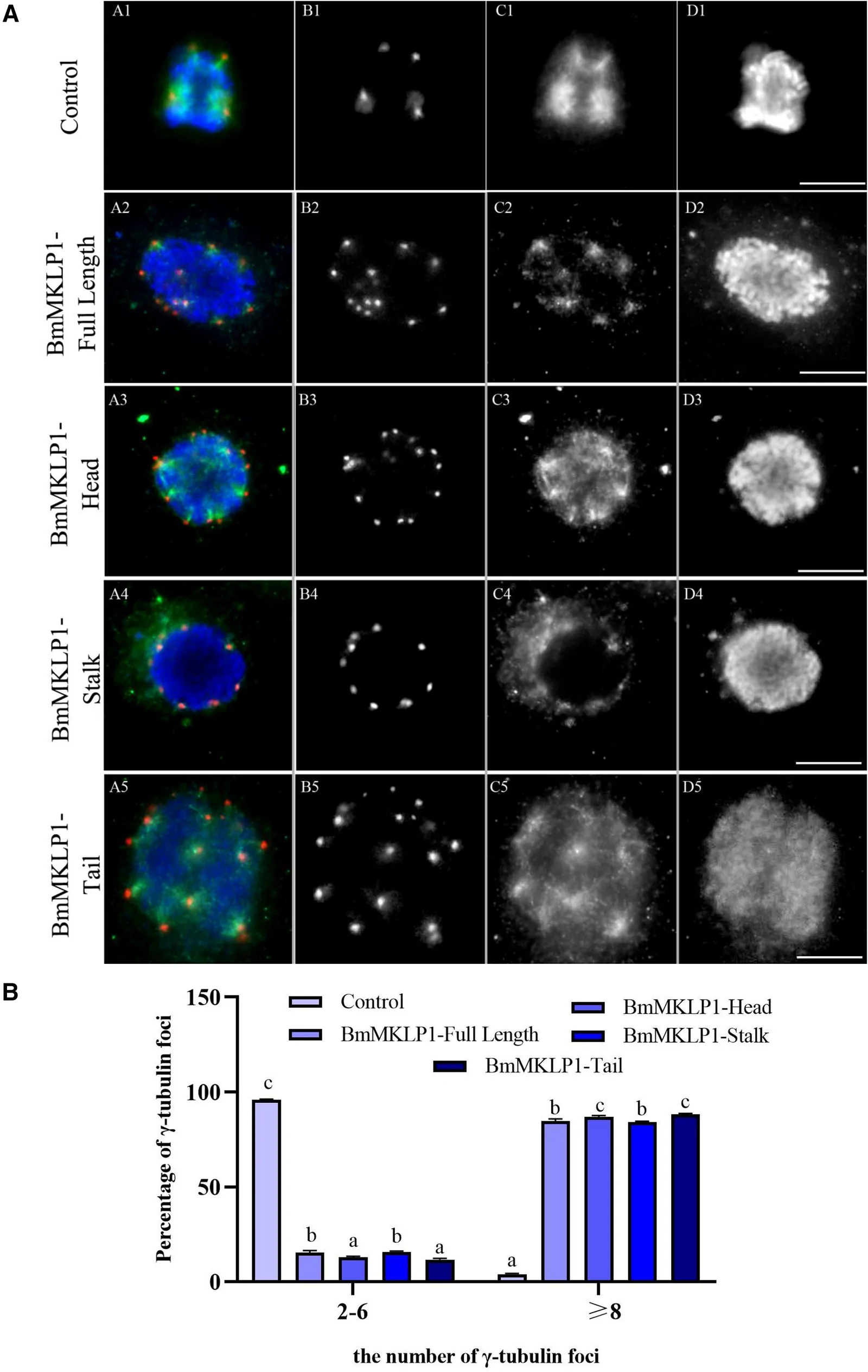
Distinct BmMKLP1 domains promote ectopic γ-tubulin recruitment and aberrant spindle organization (A) Representative immunofluorescence images of BmN4 cells in prometaphase following transient transfection with the indicated constructs. Microtubules (α-tubulin, green), microtubule-organizing centers (γ-tubulin, red), and chromatin (DAPI, blue) are shown. Merged and single-channel images illustrate aberrant spindle morphology and increased numbers of ectopic γ-tubulin foci in cells expressing the BmMKLP1 full-length, head, stalk, or tail domains compared with controls. Scale bars, 5 μm. (B) Quantification of cells containing 2–6 or ≥8 γ-tubulin foci under each condition were performed on at least 120 cells per condition from three independent experiments, expressed as a percentage of total cells. Scoring of γ-tubulin foci was conducted in a blinded manner with respect to the experimental condition. Bars represent mean ± SEM from at least three independent experiments. Different lowercase letters indicate significant differences (one-way ANOVA followed by Tukey’s HSD, *p* < 0.05).

### Expression of BmMKLP1 domains disrupts chromosome segregation fidelity

We next asked whether domain-induced spindle disorganization translated into defects in mitotic fidelity by examining β-tubulin organization and chromosome behavior in transfected BmN4 cells. In control cells, spindle architecture was preserved and chromosomes aligned and segregated normally during metaphase and anaphase. In contrast, expression of BmMKLP1-full-length significantly disrupted chromosome segregation. More strikingly, expression of any isolated BmMKLP1 domain consistently produced pronounced mitotic abnormalities, including metaphase chromosome misalignment and anaphase lagging chromosomes (Figure 5A). Quantitative analysis showed that control cells predominantly exhibited normal metaphase and anaphase configurations, whereas expression of individual BmMKLP1 domains markedly reduced the proportion of cells with intact metaphase alignment and faithful anaphase segregation, accompanied by a substantial increase in chromosomal abnormalities (Figure 5B). Together, these results indicate that disruption of spindle organization by isolated BmMKLP1 domains is associated with a severe loss of chromosome segregation fidelity, linking their shared spindle defects to genomic instability.

**Figure 5:**
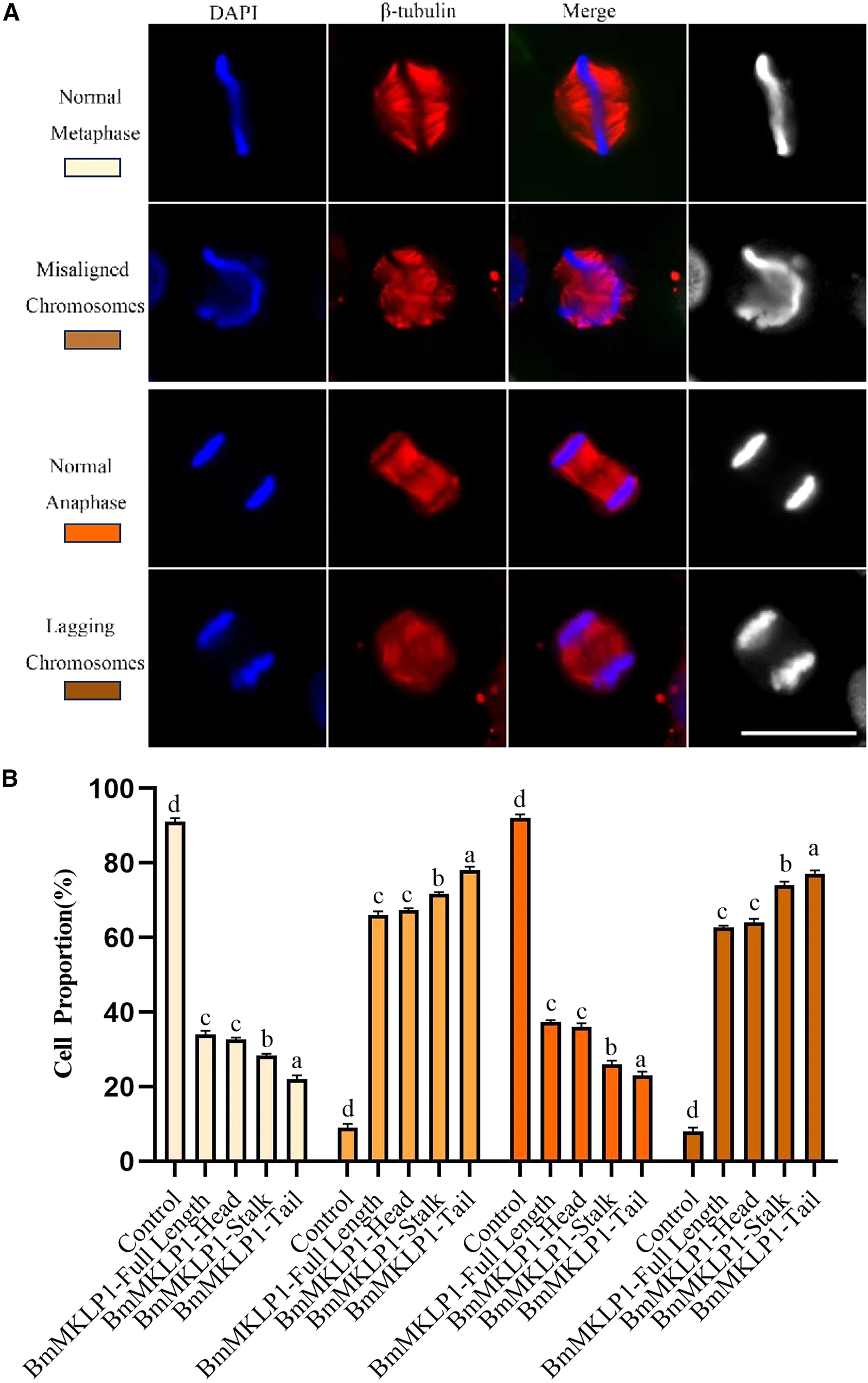
Expression of BmMKLP1 domains disrupts chromosome segregation fidelity (A) Representative immunofluorescence images of asynchronous BmN4 cells stained for DNA (DAPI, blue) and microtubules (β-tubulin, red), illustrating selected mitotic phenotypes, including normal metaphase, metaphase chromosome misalignment, normal anaphase, and lagging chromosomes. Scale bars, 5 μm. (B) Quantification of mitotic phenotypes in cells expressing individual BmMKLP1 domains. Bars represent the proportion of mitotic cells (%) exhibiting normal metaphase, metaphase chromosome misalignment, normal anaphase, or lagging chromosomes. Data are presented as mean ± SEM from three independent experiments (control, *n* = 124; BmMKLP1-full length, *n* = 147; BmMKLP1-head, *n* = 153; BmMKLP1-stalk, *n* = 168; BmMKLP1-tail, *n* = 157). Phenotype scoring was performed in a blinded fashion. Different lowercase letters indicate significant differences (one-way ANOVA followed by Tukey’s HSD, *p* < 0.05).

### BmMKLP1 domains differentially modulate cellular proliferation

Finally, we assessed the net effect of individual BmMKLP1 domains on cellular proliferation using EdU incorporation assays in BmN4 cells. Immunofluorescence analysis showed that overexpression of BmMKLP1-full-length significantly increased the proportion of EdU-positive nuclei. Expression of the stalk or tail domains also increased the proportion of EdU-positive nuclei relative to control cells, whereas expression of the head domain reduced EdU incorporation (Figure 6A). Quantitative analysis confirmed these opposing effects: full-length, stalk- and tail-expressing cells exhibited significantly higher proportions of EdU-positive nuclei, while head expression was associated with a reduction in EdU incorporation compared with controls (Figure 6B). Together, these results indicate that the BmMKLP1 domains exert divergent effects on proliferative capacity, with the full-length protein, stalk, and tail domains promoting proliferation-related DNA synthesis while the head domain suppresses it. This further highlights the functional specialization of the isolated domains, with the full-length protein phenocopying the pro-synthesis activity of the stalk and tail rather than the inhibitory effect of the head domain.

**Figure 6:**
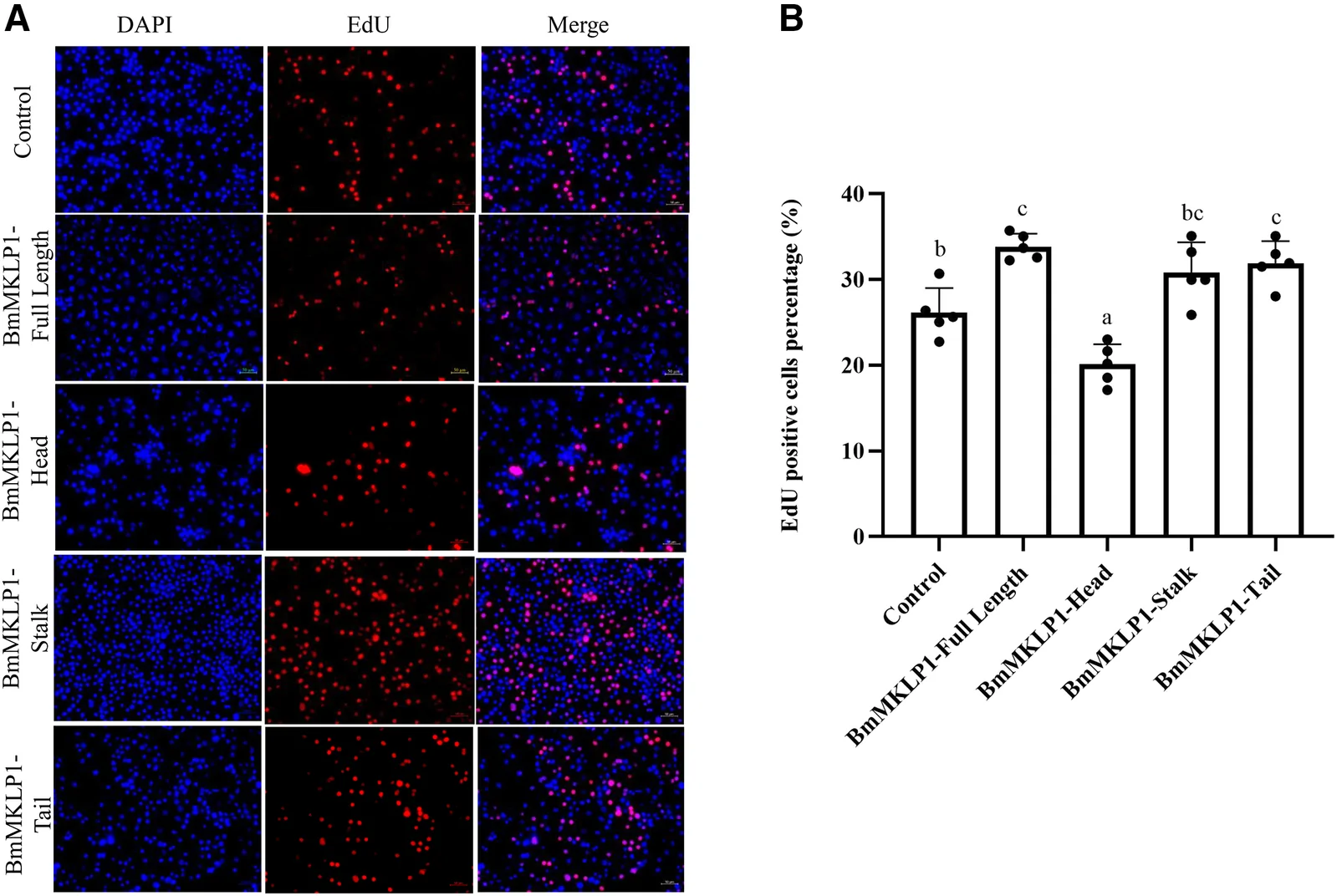
BmMKLP1 domains differentially modulate cellular proliferation (A) Representative immunofluorescence images of EdU incorporation assays showing EdU-positive nuclei (red) and total nuclei stained with DAPI (blue) in control cells and in cells expressing individual BmMKLP1 full length and different domains (head, stalk, and tail). Scale bars, 100 μm. (B) Quantification of EdU-positive cells (%). Bars represent mean ± SEM, with individual biological replicates shown as dots. Expression of the head domain is associated with reduced EdU incorporation, whereas full length, stalk-, and tail-expressing cells display increased proportions of EdU-positive nuclei. Different lowercase letters indicate significant differences (one-way ANOVA followed by Tukey’s HSD, *p* < 0.05).

## Discussion

Kinesins have traditionally been viewed as unified molecular machines, with their modular domains functioning as inseparable components of a single motor.^25^ While recent studies in kinesin-5 and kinesin-14 families have begun to highlight domain-level contributions to spindle dynamics.^14^^,^^15^^,^^16^^,^^17^^,^^18^ MKLP1 has largely remained framed as a unitary cytokinetic motor. Our work extends this view by demonstrating that *B. mori* BmMKLP1 contains individual structural domains that can function as potent, autonomous regulators when uncoupled from the full-length protein. The head, stalk, and tail domains each exert distinct effects on mitotic fidelity, ploidy, and proliferation, revealing latent activities that are normally constrained within the intact motor. These findings support a model in which BmMKLP1 operates as a modular platform whose constituent segments encode specialized regulatory capacities.

The distinct subcellular localization patterns of the isolated domains provided an early indication of their functional independence, which was conclusively demonstrated by their strikingly divergent phenotypic consequences. The head domain influenced both G2/M arrest and polyploidization, indicating that it can impact multiple cell-cycle outcomes beyond its canonical role in spindle assembly and mid-body formation.^8^^,^^26^^,^^27^ The stalk domain acted as a strong inhibitor of cell cycle progression, consistent with its central role in centralspindlin assembly.^9^^,^^28^ In contrast, the tail domain robustly promoted polyploidization, diverging from its established function in Plk1 recruitment during abscission.^29^ Notably, despite inducing G2/M arrest, the stalk and tail domains increased EdU incorporation, indicating that DNA synthesis can persist independently of mitotic progression. The nuclear enrichment of the stalk and tail domains provides a potential spatial clue for their distinct functions. The stalk’s discrete nuclear puncta, though requiring future biochemical validation to rule out non-physiological oligomerization, correlate with its potent G2/M arrest and spindle disruption, suggesting a sequestering or scaffolding role for nuclear factors; while the tail’s homogeneous nucleoplasmic distribution could facilitate broader interference with nuclear events that promote polyploidization. Together, these observations reveal BmMKLP1 as an intrinsic balancing platform that integrates competing signals governing mitotic progression and proliferative capacity.

At a mechanistic level, all three domains converged on a shared pathological activity: ectopic recruitment of γ-tubulin and disruption of spindle assembly. This convergence suggests a latent capacity for unregulated microtubule nucleation, which is consistent with sustained spindle assembly checkpoint activation underlying the G2/M arrest observed upon head and stalk expression. In contrast, the tail domain appears to bypass or override this arrest, promoting polyploidization, potentially through dominant interference with abscission or checkpoint control. While the precise molecular pathways remain to be fully delineated, we propose testable hypotheses based on domain biochemistry: the microtubule-binding head domain may sequester nucleation factors; the coiled-coil stalk might form aberrant scaffolds that concentrate γ-tubulin complexes; and the disordered tail could interfere with membrane trafficking or signaling pathways that spatially regulate γ-tubulin. This phenotype parallels multinucleation observed following MKLP1 perturbation in mammalian systems,^10^^,^^30^^,^^31^^,^^32^ but is achieved here through domain-autonomous gain-of-function rather than loss of the full-length motor.

The holocentric *B*. *mori* system was instrumental in revealing these latent activities. BmN4 cells assemble mitotic spindles through a distinctive interplay between centrosome-associated and chromatin-driven microtubule nucleation, resulting in a distributed mode of spindle organization that is particularly sensitive to disruptions in spatial control.^22^^,^^33^ As a consequence, defects that may be buffered in monocentric cells become amplified in this context, consistent with the heightened vulnerability of holocentric systems to spindle assembly defects.^34^ This intrinsic sensitivity enabled the exposure of domain-specific disruptive activities that may remain cryptic in more canonical systems, underscoring the value of holocentric models for uncovering fundamental principles of mitotic regulation.

Despite the clear domain-autonomous activities uncovered here, several limitations of this study should be acknowledged. First, our analyses rely on ectopic expression of isolated MKLP1 domains, which intentionally removes them from the regulatory constraints imposed by the full-length protein and its native interaction network. While this approach was essential to expose latent regulatory capacities, it does not directly address how these activities are coordinated or restrained within the intact MKLP1 holocomplex. Second, our work was performed in a holocentric insect cell system that is uniquely sensitive to perturbations in spindle organization and γ-tubulin regulation. Although this sensitized context enabled the detection of domain-specific activities, the extent to which similar autonomous functions operates in monocentric systems or in mammalian cells remains to be determined. Finally, while ectopic γ-tubulin recruitment emerged as a shared mechanistic consequence of domain uncoupling, the molecular intermediates linking individual MKLP1 domains to γ-tubulin complexes were not resolved. Future studies combining domain-resolved interactomics with targeted perturbations in diverse mitotic systems will be required to define how MKLP1 domains are integrated and constrained to ensure mitotic fidelity.

In summary, our systematic dissection redefines BmMKLP1 from a dedicated cytokinetic motor into a modular assembly of latent regulatory domains. The phenotypic parallels between the full-length protein and the head domain underscore the motor’s core disruptive potential, while the distinct activities of the uncoupled stalk and tail domains reveal a deeper layer of regulated functionality. These findings have broad implications for understanding kinesin evolution and regulation, suggesting that domain autonomy may represent a general strategy by which motor proteins integrate and constrain diverse regulatory functions. Future work aimed at mapping domain-specific interaction networks and testing conservation across systems will further clarify how modular kinesin regulation contributes to mitotic robustness and genomic stability across eukaryotes.

## Resource availability

### Lead contact

Further information and requests for resources and reagents should be directed to and will be fulfilled by the lead contact, Yun-Chao Kan (kanyunchao@163.com).

### Materials availability

Materials generated in this study (e.g., vector constructs) are available upon material transfer agreement (MTA) completion and upon request from the lead contact.

### Data and code availability

• Full western blot image has been deposited into Mendeley data and is publicly available as of the date of publication.
• Microscopy data reported in this paper will be shared by the lead contact upon request.
• This paper does not report original code.
• Any additional information required to reanalyze the data reported in this paper is available from the lead contact upon request.

## Acknowledgments

We extend our gratitude to all staff members and students at the Henan Key Laboratory of Insect Biology, Henan International Joint Laboratory of Insect Biology, and the College of Life Science and Agricultural Engineering at Nanyang Normal University in Henan, China. This research was funded by the Henan Provincial Natural Science Foundation—Youth Fund 252300421677, Key Research Project Plan for Higher Education Institutions in Henan province, grant number 22A180007 and special funded projects of 10.13039/501100004943Nanyang Normal University, grant number ZX2016001.

## Author contributions

Conceived and designed the experiments, B.Z. , X.-N.L., and Y.-C.K. ; performed the experiments, M.-T.S. , X.-N.L. , M.-F.Q. , S.-S.T. , and Y.L. ; analyzed the data, X.-N.L., B.Z. , and Y.-C.K. ; wrote the paper, B.Z. and A.-P.C. ; project administration, B.Z. , D.-D.L. , and L.-G.Y. ; funding acquisition, B.Z. and L.-G.Y. All authors have read and agreed to the published version of the manuscript.

## Declaration of interests

The authors declare no competing interests. The funding sources played no part in the study design, data collection, analysis, interpretation, manuscript preparation, or the decision to publish the results.

## STAR★Methods

### Key resources table

**Table:** 

REAGENT or RESOURCE	SOURCE	IDENTIFIER
**Antibodies**		

rabbit anti-γ-tubulin	Proteintech, Wuhan, China	Cat#15176-1-AP；RRID:AB_2878113
rabbit anti-β-tubulin	Proteintech, Wuhan, China	Cat#10094-1-AP；RRID:AB_2210695
mouse anti-α-tubulin	Proteintech, Wuhan, China	Cat#66031-1-Ig；RRID:AB_11042766
anti-GFP antibody	Proteintech, Wuhan, China	Cat#50430-2-AP；RRID:AB_11042881
HRP-conjugated secondary antibody	Proteintech, Wuhan, China	Cat#SA00001-7H；RRID:AB_2935611

**Bacterial strain**		

*Escherichia coli* competent cells	Thermo Fisher Scientific, Waltham, MA, USA	Cat#EC0112

**Chemicals**		

TC-100 insect medium	Livning Biotechnology, China	Cat#LVN1013
10% fetal calf serum	Thermo Fisher Scientific, Waltham, MA, USA	Cat#A5256701
EcoR*I*	Beyotime Biotechnology, China	Cat#D6329
Not*I*	Beyotime Biotechnology, China	Cat#D6497
FuGENE HD Transfection Reagent	Promega (Beijing) Biotech Co., Ltd	Cat#E2311
Paraformaldehyde	Beyotime Biotechnology, China	Cat#P0099
Triton X-100	Beyotime Biotechnology, China	Cat#ST1723
Bovine serum albumin	Beyotime Biotechnology, China	Cat#ST2249
4′,6-diamino-2-phenylindole (DAPI)	Beyotime Biotechnology, China	Cat#C1002
Propidium iodide	Beyotime Biotechnology, China	Cat#ST511
5-ethynyl-2′-deoxyuridine (EdU)	Beyotime Biotechnology, China	Cat#ST067
RIPA buffer	Beyotime Biotechnology, China	Cat#P0013B

**Critical commercial assays**		

In-Fusion HD Cloning Kit	Takara, Dalian, China	Cat#639650
Click-iT EdU Alexa Fluor™ 594 Imaging Kit	Thermo Fisher Scientific, Waltham, MA, USA	Cat#C10339
BCA Protein Assay Kit	Thermo Fisher Scientific, Waltham, MA, USA	Cat#23227
ECL Western Blot Substrates	Thermo Fisher Scientific, Waltham, MA, USA	Cat#32209

**Experimental models: Cell lines**		

BmN4	Kan Lab	NA

**Oligonucleotides**		

Primers used for vector constructions	This manuscript	See Table S1

**Recombinant DNA**		

pIZT/V5-His	Invitrogen – Thermo Fisher Scientific, Waltham, MA, USA	Cat#V801001
pIZT/V5-GFP/BmMKLP1-Head	This study	NA
pIZT/V5-GFP/BmMKLP1-Stalk	This study	NA
pIZT/V5-GFP/BmMKLP1-Tail	This study	NA
pIZT/V5-GFP/BmMKLP1-Full Length	This study	NA

**Software and algorithms**		

GraphPad Prism v9.0	NA	https://www.graphpad.com
MARCOIL	Gabler et al.^23^	https://toolkit.tuebingen.mpg.de/tools/marcoil
IUPred2A	Erdős & Dosztányi^24^	https://iupred2a.elte.hu/
NCBI MSA Viewer 1.26	NA	https://www.ncbi.nlm.nih.gov/projects/msaviewer/
FlowJo v10.5.3	NA	https://flowjo.com/flowjo/download

### Experimental model and study participant details

#### Cell line

Silkworm ovary-derived BmN4 cells were cultured in TC-100 insect medium (BBI Life Sciences, Shanghai, China) supplemented with 10% fetal calf serum (Gibco, Invitrogen, Shanghai, China) at 27°C. The cell line was authenticated by morphological observation and tested negative for mycoplasma contamination using PCR-based detection method.

### Method details

#### Plasmid construction

The three operational domains of BmMKLP1 were amplified from a BmN4 cell cDNA library. Domain boundaries were defined based on annotated BmMKLP1 architecture (GenBank: XP_037875228.1) and coiled-coil predictions in MARCOIL (https://toolkit.tuebingen.mpg.de/tools/marcoil), yielding a Head fragment (aa 28–429), a central Stalk fragment (aa 502–662), a Tail fragment (aa 663–960) and a full-length fragment (aa 28–960). The pIZT/V5-His vector (Invitrogen, Carlsbad, CA, USA) was modified by excising the *GFP* coding sequence to generate the pIZT/V5-zb1 backbone. The GFP coding sequence was amplified from pIZT/V5-His using specific primers (Table S1). Each BmMKLP1 domain was fused to *GFP* amplicon via overlap PCR using primers containing complementary overhangs (Table S1). The resulting fusion fragments (GFP/BmMKLP1-Head, GFP/BmMKLP1-Stalk, GFP/BmMKLP1-Tail, and GFP/BmMKLP1-Full Length) were digested with EcoR*I* and Not*I* and inserted into the corresponding sites of the multicloning site region of pIZT/V5-zb1 and in a transcriptional fusion to the OpIE2 promoter for constitutive expression using the In-Fusion HD Cloning Kit (639650; Takara, Dalian, China). The four constructs (pIZT/V5-GFP/BmMKLP1-Head; pIZT/V5-GFP/BmMKLP1-Stalk; pIZT/V5-GFP/BmMKLP1-Tail; pIZT/V5-GFP/BmMKLP1-Full Length) were validated by restriction digestion and Sanger sequencing. The verification data, including representative gel images and sequencing chromatograms, are provided in Figures S1–S4.

#### Cell culture and transfection

For experiments, BmN4 cells were seeded into 12-well plates at a density of 2 × 10^5^ cells per well. After 24 h, cells were transfected with 0.5 μg of plasmid BmMKLP1- Full Length, Head, -Stalk, -Tail, or empty vector control (CK) using FuGENE HD Transfection Reagent (Invitrogen, Shanghai, China) according to the manufacturer’s instructions. Cells were harvested 72 h post-transfection for downstream analysis.

#### Immunofluorescence and microscopy

For immunofluorescence analysis, transfected BmN4 cells grown on glass coverslips were fixed with 4% paraformaldehyde for 10 min at room temperature (RT), permeabilized with 0.5% Triton X-100 for 5 min, and blocked with 3% bovine serum albumin (BSA) for 30 min. Primary antibodies diluted in 3% BSA were applied overnight at 4°C. The following antibodies were used: rabbit anti-γ-tubulin (1:1000; Proteintech, Wuhan, China; 15176-1-AP), rabbit anti-β-tubulin (1:1000; Proteintech, Wuhan, China; 10094-1-AP), and mouse anti-α-tubulin (1:500; Proteintech, Wuhan, China; 66031-1-Ig). These antibodies have been previously validated for use in BmN4 cells^22^(Zhang et al., 2024). After three washes with phosphate-buffered saline (PBS; 137 mM NaCl, 2.7 mM KCl, 10 mM Na_2_HPO_4_, 1.8 mM KH_2_PO_4_, pH 7.4), cells were incubated for 2 h at 37°C with Alexa Fluor-conjugated secondary antibodies (goat anti-mouse 488 or goat anti-rabbit 594; 1:1000; Jackson ImmunoResearch, PA, USA). DNA was stained with 4′,6-diamino-2-phenylindole (DAPI; 0.01 mg/mL) for 5 min at RT. Coverslips were mounted using a DABCO-based antifade mounting medium and sealed. Images were acquired using a ZEISS Axio Observer 7 inverted fluorescence microscope (Carl Zeiss AG, Oberkochen, Germany) equipped with appropriate filter sets. For quantitative comparisons, images were acquired using identical exposure settings across samples. The scoring was performed in a blinded manner with respect to the experimental condition. Specifically, all images were assigned random codes by a second researcher prior to analysis, and the investigator performing the quantification was unaware of the sample group identity until after all counts and classifications were completed. A minimum of 120 cells per condition from at least 3 independent experiments were analyzed for statistical significance.

#### Flow cytometry for cell cycle and polyploidy analysis

Cells harvested 72 h post-transfection were washed three times with ice-cold PBS, and fixed in 75% ethanol at 4°C for 24 h. Fixed cells were washed twice with PBS to remove ethanol, treated with RNase A (100 μg/mL) for 30 min at 37°C, and stained with propidium iodide (PI, 50 μg/mL in PBS with 0.1% Triton X-100) for 30 min at 37 °C in the dark. Stained cell suspensions were filtered through a 40 μm nylon mesh. Samples were analyzed on a CytoFLEX flow cytometer (Beckman Coulter, Brea, CA, USA), collecting a minimum of 10,000 single-cell events per sample. PI was excited with a 488 nm laser, and fluorescence was detected using a 610/20 nm bandpass filter. For flow cytometry analyses, GFP-positive cells were gated based on a fluorescence intensity threshold determined using untransfected control cells from the same experiment. This gating strategy specifically isolates the transfected population for analysis, ensuring that the measured cell cycle and polyploidy profiles are derived from cells expressing the GFP-tagged domains. Cell cycle distribution (G0/G1, S, G2/M) and the proportion of polyploid cells (>4 N DNA content) were determined by modeling PI fluorescence intensity histograms with the Dean-Jett-Fox algorithm implemented in FlowJo software (version 10.5.3).

#### EdU proliferation assays

Cell proliferation was assessed 72 h post-transfection using a Click-iT EdU Alexa Fluor 594 Imaging Kit (Thermo Fisher Scientific, Waltham, MA, USA). Cells were pulsed with 10 μM 5-ethynyl-2′-deoxyuridine (EdU) for 2 h at 27°C and then fixed with 4% paraformaldehyde in PBS for 15 min at RT. After permeabilization with 0.1% Triton X-100 in PBS for 15 min at RT, the Click-iT reaction cocktail was prepared according to the manufacturer’s instructions and incubated with the samples for 30 min at RT in the dark. Following the Click-iT reaction, cells were washed once with 3% BSA in PBS. DNA was counterstained with DAPI (1 μg/mL in PBS) for 30 min at RT, followed by two washes with PBS. Images were acquired using a ZEISS Axio Observer 7 inverted fluorescence microscope (Carl Zeiss AG). The percentage of EdU-positive cells was quantified by manually counting at least 500 cells per condition across three independent experiments.

#### Western blot

Cells were harvested 72 h post-transfection and lysed in RIPA buffer (Beyotime Biotechnology, China) supplemented with 1 mM phenylmethylsulfonyl fluoride (PMSF) and Halt Protease Inhibitor Cocktail, EDTA-Free (Thermo Fisher Scientific, Waltham, MA, USA). Total protein concentration was determined using a BCA Protein Assay Kit (Thermo Fisher Scientific, Waltham, MA, USA). Equal amounts of protein (20 μg) were resolved by 10% SDS-PAGE and transferred to PVDF membranes (Millipore, Burlington, MA, USA). Membranes were blocked in 5% non-fat milk in TBST (Tris-buffered saline with 0.1% Tween 20) for 1 h at RT and incubated overnight at 4°C with anti-GFP antibody (1:1000, Proteintech, Wuhan, China; 50430-2-AP). After washing, membranes were incubated with HRP-conjugated secondary antibody (1:5000, Proteintech, SA00001-7H) for 1 h at RT. Blots were developed using ECL chemiluminescence (Thermo Fisher Scientific, Waltham, MA, USA) and imaged using a ChemiDoc imaging system (Bio-Rad, Hercules, CA, USA).

#### Statistical analysis

Statistical analyses were performed using GraphPad Prism software (v9.0, GraphPad Software, San Diego, CA, USA). Data normality and homogeneity of variances were assessed using the Shapiro-Wilk and Brown-Forsythe tests, respectively. For comparisons involving more than two experimental groups, one-way analysis of variance (ANOVA) followed by Tukey’s honestly significant difference (HSD) post hoc test was applied. Data are presented as mean ± standard error of the mean (SEM) from at least three independent biological replicates. A *p*-value of less than 0.05 was considered statistically significant.
