# Supplementary material for: Domain-autonomous MKLP1 modules divergently regulate mitotic fidelity and polyploidy in a holocentric system
Source: iScience. 2026 Oct 1;29(10):117149. doi: 10.1016/j.isci.2026.117149 (PMC13657243; doi:10.1016/j.isci.2026.117149)
Supplement: Supplementary file 1 [file mmc1.pdf]

iScience, Volume *29*

Supplemental information

Domain-autonomous MKLP1 modules divergently regulate mitotic fidelity and polyploidy in a holocentric system

Bing Zhang, Xiao-Ning Liu, Mei-Ting Song, Meng-Fan Qin, Shu-Shu Tian, Yu Lin, Dan-Dan Li, Lun-Guang Yao, Camilo Ayra-Pardo, and Yun-Chao Kan

Supplementary Figures

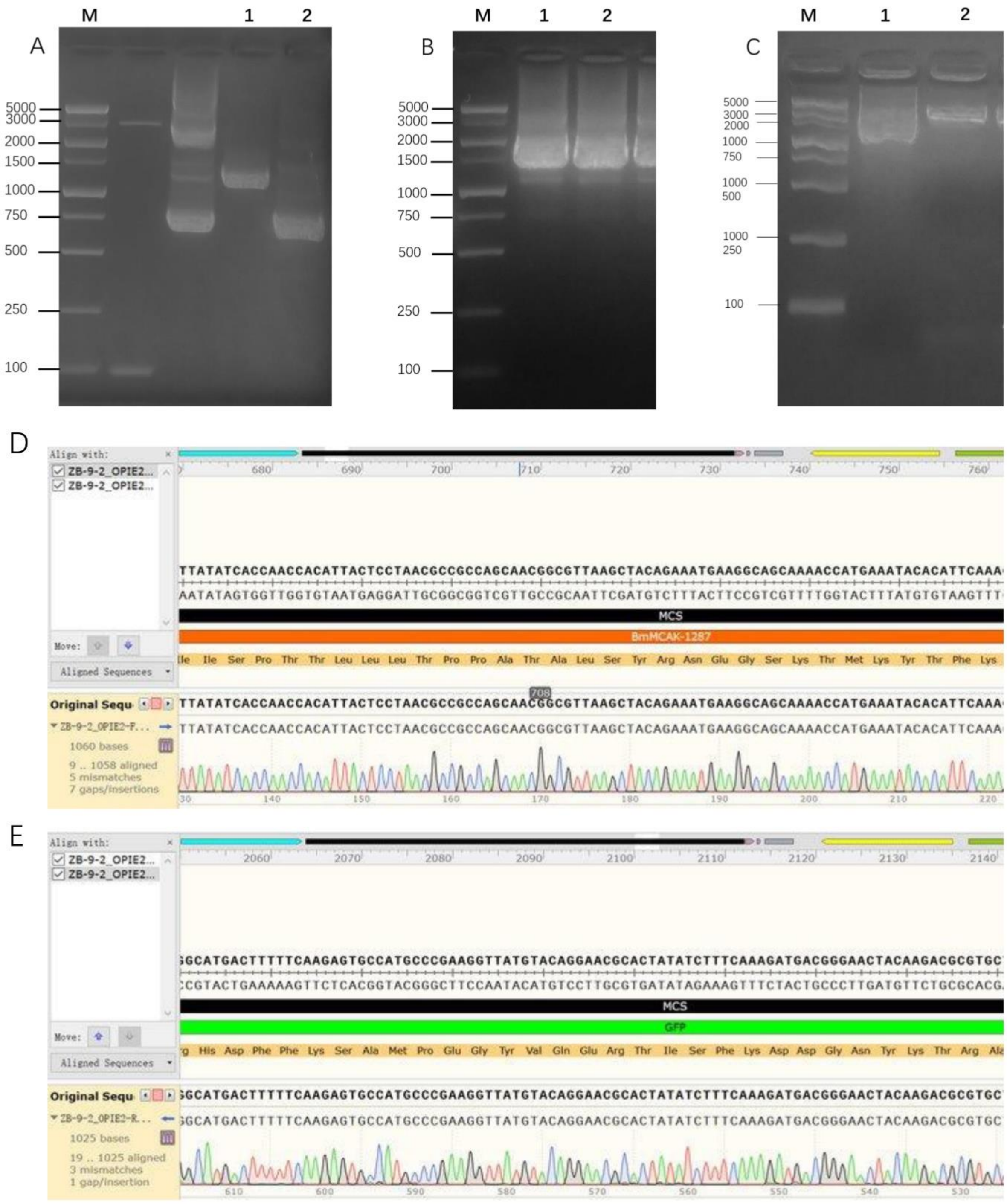

Figure S1: Plasmid construction and sequencing validation of BmMKLP1-Head (pIZT/V5-His-BmMKLP1-Head-GFP)

(A) Amplification of gene fragments: Lane M, DNA molecular weight marker with indicated sizes; Lane 1, BmMKLP1-Head fragment; Lane 2, GFP fragment. **(B)** Fusion PCR amplification of the BmMKLP1-Head-GFP fragment using overlap extension PCR; Lane M, DNA molecular weight marker. **(C)** Restriction enzyme digestion of the pIZT/V5-His-zb1 vector: Lane M, DNA molecular weight marker; Lane 1, undigested control plasmid; Lane 2, digested plasmid. **(D, E)** Sanger sequencing verification of the constructed pIZT/V5-His-BmMKLP1-Head-GFP plasmid.

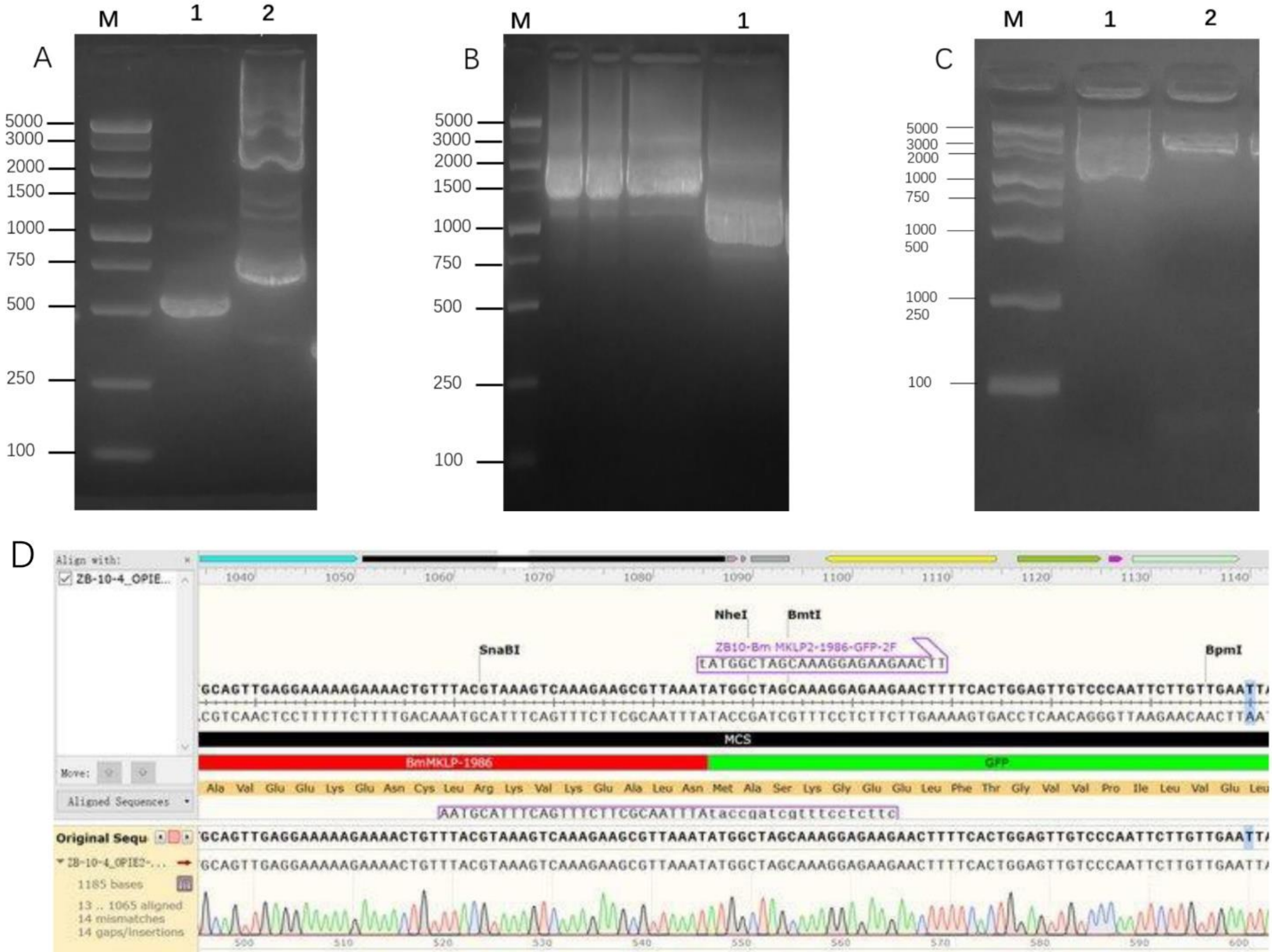

Figure S2: Plasmid construction and sequencing validation of BmMKLP1-Stalk (pIZT/V5-His-BmMKLP1-Stalk-GFP)

(A) Amplification of gene fragments: Lane M, DNA molecular weight marker with indicated sizes; Lane 1, BmMKLP1- Stalk fragment; Lane 2, GFP fragment. **(B)** Fusion PCR amplification of the BmMKLP1- Stalk -GFP fragment using overlap extension PCR; Lane M, DNA molecular weight marker. **(C)** Restriction enzyme digestion of the pIZT/V5-His-zb1 vector: Lane M, DNA molecular weight marker; Lane 1, undigested control plasmid; Lane 2, digested plasmid. **(D)** Sanger sequencing verification of the constructed pIZT/V5-His-BmMKLP1- Stalk -GFP plasmid.

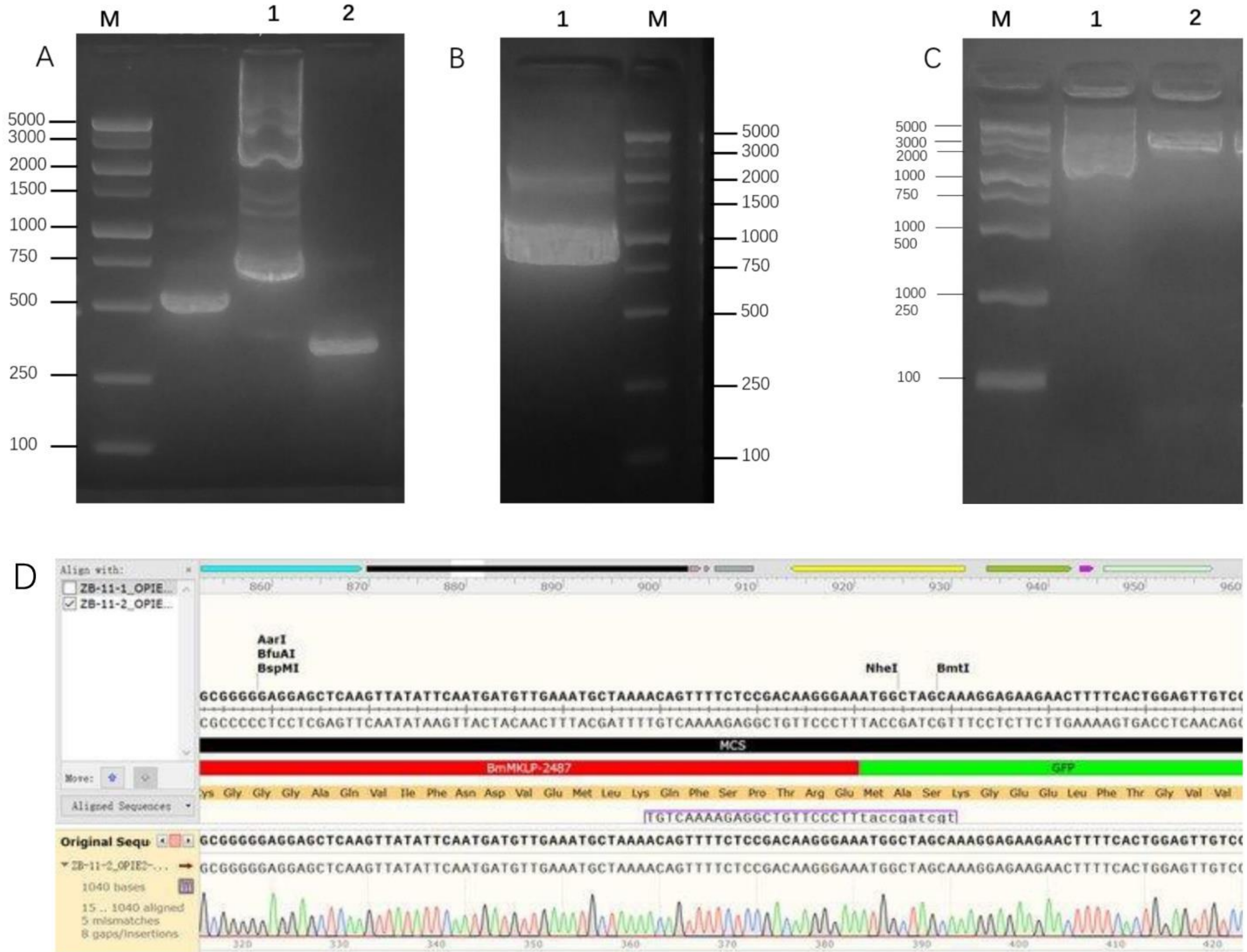

Figure S3: Plasmid construction and sequencing validation of and BmMKLP1-Tail (pIZT/V5-His- BmMKLP1-Tail-GFP)

(A) Amplification of gene fragments: Lane M, DNA molecular weight marker with indicated sizes; Lane 1, GFP fragment; Lane 2, BmMKLP1- Tail fragment. **(B)** Fusion PCR amplification of the BmMKLP1- Tail -GFP fragment using overlap extension PCR; Lane M, DNA molecular weight marker. **(C)** Restriction enzyme digestion of the pIZT/V5-His-zb1 vector: Lane M, DNA molecular weight marker; Lane 1, undigested control plasmid; Lane 2, digested plasmid. **(D)** Sanger sequencing verification of the constructed pIZT/V5-His-BmMKLP1- Tail -GFP plasmid.

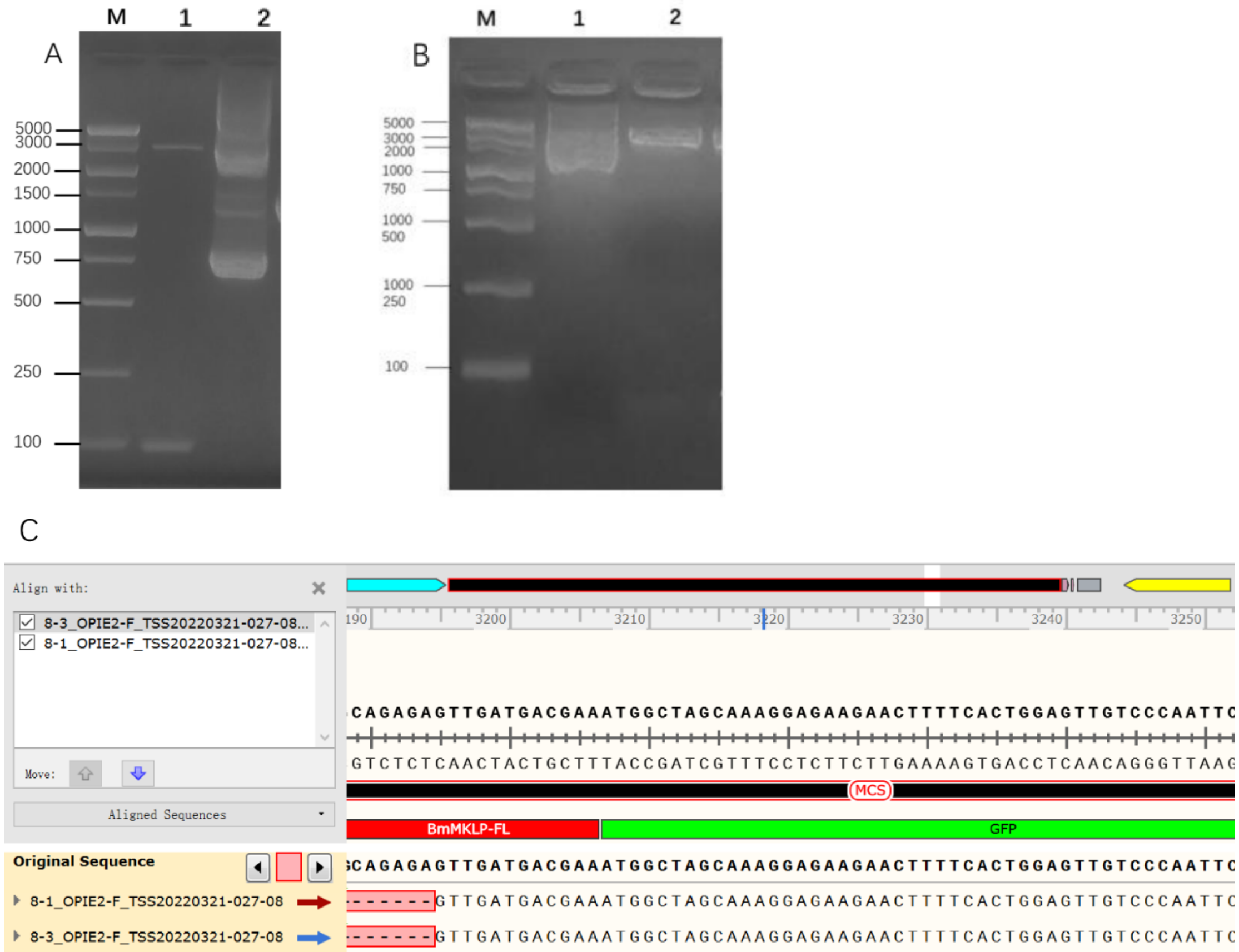

Figure S4: Plasmid construction and sequencing validation of and BmMKLP1-Full Length (pIZT/V5-His- BmMKLP1- Full Length -GFP)

(A) Amplification of gene fragments: Lane M, DNA molecular weight marker with indicated sizes; Lane 1, Fusion PCR amplification of the BmMKLP1- Full Length -GFP fragment using overlap extension PCR; Lane 2, BmMKLP1- Full Length fragment. (B) Restriction enzyme digestion of the pIZT/V5-His-zb1 vector: Lane M, DNA molecular weight marker; Lane 1, undigested control plasmid; Lane 2, digested plasmid. (D) Sanger sequencing verification of the constructed pIZT/V5-His-BmMKLP1- Full Length -GFP plasmid.

Table S1. Primers utilized in this study.

Primer	PriPrimer sequence (5'-3')	Purpose
pIZT/V5-zb1-F	CGAATTTAAAGCTTGGTACCGAG	Plasmid construction
pIZT/V5-zb1-R	ATCCAGACATGATAAGATACATTGATGA	
BmMKLP1-Head-1F	tagtccagtgtggtggaattcGATCCTGTACAAGTCTACTGTAGACTTCG	
BmMKLP1-Head -1R	tgctagccatTGCCATCTCAGCAAATCTCATC	
BmMKLP1-Head-GFP-2F	tgagatggcaATGGCTAGCAAAGGAGAAGAACTT	
BmMKLP1-Stalk-1F	tagtccagtgtggtggaattcATTAAGGAACTGATGGCACATTTAGA	
BmMKLP1-Stalk-1R	cttctcctttgctagccatATTTAACGCTTCTTTGACTTTACGTAA	
BmMKLP1-Stalk-GFP-2F	tATGGCTAGCAAAGGAGAAGAACTT	
BmMKLP1-Tail-1F	tagtccagtgtggtggaattcGCGAATCCACGCCATAGACG	
BmMKLP1-Tail-1R	tgctagccatTTCCCTTGTCGGAGAAAACTGT	
BmMKLP1-Tail-GFP-2F	gacaagggaaATGGCTAGCAAAGGAGAAGAACTT	
BmMKLP1-Full Length -1F	tagtccagtgtggtggaattcATGAAGTCCGTTAAATCGAAACAGA	
BmMKLP1-Full Length -1R	cctttgctagccatTTCGTCATCAACTCTCTGCTTCTT	
BmMKLP1-Full Length -GFP-2F	gacgaaATGGCTAGCAAAGGAGAAGAACTT	
EGFP-2F	cggacaacgaatatATGGCTAGCAAAGGAGAAGAACTT	
EGFP-2R	gccctctagactcgagcggccgcATCCATGCCATGTGTAATCCCA	

* Complementary overhang sequences used for overlap PCR cloning are shown in lowercase letters.
